# Tubular Elabela-APJ axis attenuates ischemia-reperfusion induced acute kidney injury and the following AKI-CKD transition by protecting renal microcirculation

**DOI:** 10.7150/thno.84308

**Published:** 2023-06-04

**Authors:** Mingrui Xiong, Hong Chen, Yu Fan, Muchuan Jin, Dong Yang, Yuchen Chen, Yu Zhang, Robert B. Petersen, Hua Su, Anlin Peng, Congyi Wang, Ling Zheng, Kun Huang

**Affiliations:** 1 Tongji School of Pharmacy, Tongji Medical College and State Key Laboratory for Diagnosis and Treatment of Severe Zoonotic Infectious Diseases, Huazhong University of Science & Technology, Wuhan, China, 430030.; 2 Hubei Key Laboratory of Cell Homeostasis, Frontier Science Center for Immunology and Metabolism, College of Life Sciences, Wuhan University, Wuhan, China, 430072.; 3 Foundational Sciences, Central Michigan University College of Medicine, Mt. Pleasant, MI, USA, 48859.; 4 Department of Nephrology, Union Hospital, Tongji Medical College, Huazhong University of Science and Technology, Wuhan, China, 430030.; 5 Department of Pharmacy, The Third Hospital of Wuhan, Tongren Hospital of Wuhan University, Wuhan, China, 430075.; 6 The Center for Biomedical Research, Department of Respiratory and Critical Care Medicine, NHC Key Laboratory of Respiratory Disease, Tongji Hospital, Tongji Medical College, Huazhong University of Science & Technology, Wuhan, China, 430030.

**Keywords:** elabela, APJ, acute kidney injury, AKI-CKD transition, renal microvascular blood flow

## Abstract

**Rationale:** Ischemia-reperfusion injury (I/R) is a common cause of acute kidney injury (AKI). Post-ischemic recovery of renal blood supply plays an important role in attenuating injury. Exogenous application of elabela (ELA) peptides has been demonstrated by us and others to alleviate AKI, partly through its receptor APJ. However, the endogenous role of ELA in renal I/R remains unclear.

**Methods:** Renal tubule specific ELA knockout (*Apela^Ksp^* KO) mice challenged with bilateral or unilateral I/R were used to investigate the role of endogenous ELA in renal I/R. RNA-sequencing analysis was performed to unbiasedly investigate altered genes in kidneys of *Apela^Ksp^* KO mice. Injured mice were treated with ELA32 peptide, Nω-hydroxy-nor-L-arginine (nor-NOHA), prostaglandin E2 (PGE2), Paricalcitol, ML221 or respective vehicles, individually or in combination.

**Results:** ELA is mostly expressed in renal tubules. Aggravated pathological injury and further reduction of renal microvascular blood flow were observed in *Apela^Ksp^* KO mice during AKI and the following transition to chronic kidney disease (AKI-CKD). RNA-seq analysis suggested that two blood flow regulators, arginine metabolizing enzyme arginase 2 (ARG2) and PGE2 metabolizing enzyme carbonyl reductases 1 and 3 (CBR1/3), were altered in injured *Apela^Ksp^* KO mice. Notably, combination application of an ARG2 inhibitor nor-NOHA, and Paricalcitol, a clinically used activator for PGE2 synthesis, alleviated injury-induced AKI/AKI-CKD stages and eliminated the worst outcomes observed in *Apela^Ksp^* KO mice. Moreover, while the APJ inhibitor ML221 blocked the beneficial effects of ELA32 peptide on AKI, it showed no effect on combination treatment of nor-NOHA and Paricalcitol.

**Conclusions:** An endogenous tubular ELA-APJ axis regulates renal microvascular blood flow that plays a pivotal role in I/R-induced AKI. Furthermore, improving renal blood flow by inhibiting ARG2 and activating PGE2 is an effective treatment for AKI and prevents the subsequent AKI-CKD transition.

## Introduction

Acute kidney injury (AKI), associated with high mortality, is defined by a rapid increase in serum creatinine, decreased urine output, or both 1, 2. Although considered to be a reversible disease, AKI patients have a higher risk of transition to chronic kidney disease (CKD) 3. Ischemia-reperfusion injury (I/R) is the most common cause of AKI and occurs in multiple clinical settings including cardiac surgery, major surgery, or cardiorenal syndrome 4. The pathophysiological mechanisms of I/R-induced AKI, as well as the AKI to CKD transition, remain poorly understood, and effective therapy to treat/prevent AKI is lacking.

The kidney is one of the most vascularized organs receiving 20-25% of the cardiac output in adults 5. When the renal artery supplies blood to the kidney, it forms a complex and dynamic microcirculatory network that provides intraglomerular pressure, peritubular capillary pressure, perfusion and oxygenation to sustain renal function 6. This vascular microcirculation network, which is highly sensitive to hypoxic conditions, is a major target in I/R 7, 8.

Apelin receptor (APJ), a membrane G protein-coupled receptor (GPCR), plays crucial roles in the regulation of cardiac contractility and blood pressure 9. Elabela (ELA, encoded by *APELA*) is an endogenous ligand of APJ receptor 10, 11, but it may also function independent of APJ 10, 11. ELA is synthesized as a 54-residue pro-peptide that is cleaved to produce a mature 32-residue secreted peptide, ELA32 10-13.

As a physiological regulator, ELA was first identified as an essential peptide for cardiogenesis in zebrafish 10, 11, and later as a vasodilatory agent and positive inotrope in rats 13, 14. ELA was found to be downregulated in the lung of pulmonary arterial hypertension (PAH) patients, which was attenuated by administrating exogenous ELA 13. We previously demonstrated that exogenous ELA32 and its analogue peptides protect against I/R-induced AKI 12, 15. However, the role of endogenous ELA in I/R-induced AKI and the following transition to CKD remains unknown.

In the current study, renal tubule specific *Apela* knockout (*Apela^Ksp^* KO) mice were challenged with I/R. *Apela^Ksp^* KO mice showed more aggravated pathologies at the AKI and AKI-CKD transition stages, with increased inflammation and fibrosis observed at the AKI-CKD transition. RNA-sequencing suggested dramatically altered expression of genes that regulate blood flow including arginase 2 (*ARG2*) and prostaglandin E2 (PGE2) regulating enzymes carbonyl reductase 1/3 (*CBR1*/*CBR3*). Furthermore, aggravated renal microvascular injury was found in injured *Apela^Ksp^* KO mice at both stages. In C57BL/6 and *Apela^Ksp^* KO mice, treatments with an ARG2 inhibitor, nor-NOHA, and a clinically used PGE2 synthesis activator, Paricalcitol, either separately or in combination, alleviated I/R-induced AKI; while combination treatment alleviated AKI-CKD. Moreover, an APJ inhibitor ML221 blocked the beneficial effects of ELA32 peptide on AKI. Together, our results reveal a novel role of the tubular ELA-APJ axis in regulating renal microvasculature and suggest a potential therapeutic approach for preventing AKI and the subsequent AKI-CKD transition.

## Materials and Methods

### Animals

*Apela^flox/flox^* mice in C57BL/6 background were generated by Cyagen Biosciences (Suzhou, Jiangsu, China). *Ksp*-Cre female mice (JAX, No.012237) (expressing Cre under the control of *Ksp*-*cadherin* promoter which is expressed exclusively in renal tubular epithelial cells of mouse 16) in C57BL/6 background were crossing with *Apela^flox/flox^* male mice to generate renal tubular cell specific ELA KO (*Apela^Ksp^* KO) mice. Age-matched male *Apela^Ksp^* KO mice and *Apela^flox/flox^ Cre^-/-^* (wildtype, WT) littermates were used. Expected Mendelian ratio of mice with different genotypes were found in this study. Male C57BL/6 mice (8-week-old, 25 ± 3 g) were obtained from the Hubei Center for Disease Control and Prevention. Animals were handled according to the Guidelines of the China Animal Welfare Legislation, as approved by the Committee on Ethics in the Care and Use of Laboratory Animals, College of Life Sciences, Wuhan University.

### AKI models and treatments

To induce AKI, a bilateral I/R (bI/R) model was used. Briefly, mouse was anesthetized and underwent midline abdominal incisions, both kidneys were clamped to block blood flow for 30 minutes. After ischemia, clamps were released to start reperfusion. To study the development from AKI to CKD, unilateral renal I/R (uI/R) was used as we previously described 12, 17. Briefly, left renal pedicle of mouse was bluntly clamped for 45 minutes, reperfusion was achieved by removing the clamp. Sham operated mice (Sham) were used as respective controls. Mice were sacrificed at day 2 or 3 after bI/R (bI/R 2D or bI/R 3D); or at day 1, 3, 7 and 21 after unilateral I/R (uI/R 1D, uI/R 3D, uI/R 7D, and uI/R 21D). Death of some animals were observed at bI/R 3D, but not during the uI/R study.

For treatments, all the drugs used in the present study were intraperitoneally injected at the time and dosages used as previously reported 12, 18-21. *N*^ω^-hydroxy-nor-L-arginine (nor-NOHA; 50 mg/kg body weight; Bachem, Budendorf, Switzerland; dissolved in sterilized saline) was administrated one time right after reperfusion, while Paricalcitol (0.3 μg/kg body weight; Hengrui Med., Jiangsu, China; diluted by sterilized saline) was injected one time at 1 day before the injury. PGE2 (80 μg/kg body weight; D133402, Aladdin, Shanghai, China) and ML221 (10 mg/kg body weight; T4390, Topscience, Shanghai, China) (both dissolved in 90% sterilized saline and 10% DMSO) were administrated every other day from the day of the injury (right after reperfusion) to the end of the experiment. ELA32 peptide (300 pM/kg body weight; Genscript Biotech., Nanjing, China; dissolved in sterilized saline) was administrated twice per day from the day of the injury (right after reperfusion) to the end of the experiment. All treatments were administrated individually or in combination as indicated (Vehicle, injured mice treated with vehicle; nor-NOHA, injured mice treated with nor-NOHA; PGE2, injured mice treated with PGE2; Pari, injured mice treated with Paricalcitol; nor-NOHA+PGE2, injured mice treated with nor-NOHA and PGE2; nor-NOHA+Pari, injured mice treated with nor-NOHA and Paricalcitol; Saline, injured mice treated with sterilized saline; E32, injured mice treated with ELA32 peptide; ML221, injured mice treated with ML221; E32+ML221, injured mice treated with ELA32 peptide and ML221; nor-NOHA+Pari+ML221, injured mice treated with nor-NOHA, Paricalcitol and ML221).

### Assessment of renal function and renal PGE2

Renal function was evaluated by measuring serum creatinine and BUN (blood urea nitrogen) levels using a creatinine reagent kit and a BUN reagent kit, respectively (both from Jiancheng Bio., Nanjing, China). Renal PGE2 level was measured using a mouse PGE2 ELISA kit (HuaMei Biotech., Wuhan, China).

### Antibody for ELA

A polyclonal antibody against ELA was raised by immunizing rabbit with the C-terminal region of ELA peptide (Dia-An Biotech. Wuhan, China), and its specificity on mouse renal sections was demonstrated (Figure S1).

### Renal histology evaluation, immunohistochemical and immunofluorescent staining

Paraffined embedded sections were used for H&E, Sirius Red and immunohistochemical staining. For pathological evaluation, H&E stained renal sections were assessed and evaluated as previously described 12, 22. For evaluation of fibrosis, a Sirius Red staining kit (SenBeijia Biotech., Nanjing, China) was used. For immunohistochemical staining, primary antibodies for Ly6G, CD3, F4/80 or CD31 (information listed in Table S1) were applied overnight at 4 °C. Sections were then incubated with respective biotinylated secondary antibody, followed by incubation in ABC-peroxidase solution (Vector laboratories, Burlingame, CA), and visualized using 3,3′-diaminobenzidine (DAB, CWbiotech, Beijing, China). Quantitative analysis of positively stained cells was performed as previously reported 23, 24.

Immunofluorescent staining was performed on renal cryosections as previously reported 25, 26. ARG2 antibody (information listed in Table S1) or a homemade ELA antibody was applied, and sequentially incubated with respective Alexa Fluor-labeled secondary antibody (Invitrogen, Carlsbad, CA). Sections then were covered with DAPI (Sigma) and anti-fading medium (Invitrogen). PNA (peanut agglutinin) and DBA (dolichos biflorus agglutinin) was used to identify the distal tubules and collecting duct, while LTL (lotus tetragonolobus lectin) was used to identify the proximal tubules. Imaging was taken with a TCS SP8 confocal microscope (Leica, German).

### RNA sequencing and analysis

Total renal RNA was isolated. RNA sequencing and data analyses were performed using Novogene Bioinformatics (Beijing, China) as previously reported 27, 28. Differentially expressed genes were identified with threshold of adjusted p-value < 0.05 and |log2FoldChange| > 0. Sequencing dataset described in this work is available in NCBI Gene Expression Omnibus (GSE 213299; reviewer token oziraaiwllqpbed).

### Quantitative real-time PCR (qPCR) and Western blots

qPCR and Western blots were performed as previously described 29, 30. Antibodies and primers used are provided in Tables S1-2.

### Microvascular flow measurements

Renal microvascular flow was assessed using the laser speckle contrast approach. Briefly, the left kidney was exposed and scanned with a moorFLPI-2 blood flow imager and renal microcirculation was quantified using the imager's analysis software (Moor Instruments, Wilmington, DE). Throughout the measurement, mice were under anesthesia using 1.5% isoflurane. Core body temperature was monitored and maintained at 37.2 ± 0.1°C.

### Statistical analyses

Data are presented as mean ± SD. The normal distribution of data was tested by GraphPad Prism 8. For two-group comparison, normally distributed data was analyzed using unpaired two-tailed Student's test, while non-normally distributed data was analyzed using Mann-Whitney U test. For multiple-group comparison of normally distributed data, one-way ANOVA with Tukey's test was used for a single independent variable, while two-way ANOVA with Tukey's test was used for two independent variables. Differences were considered statistically significant at *P* < 0.05.

## Results

### Tubular ELA is downregulated during renal I/R-induced AKI

Previously, we reported that *Apela* is downregulated at three days after injury in a mouse unilateral renal I/R model 12. To obtain a clearer picture about injury-induced alteration of ELA during AKI and the following AKI-CKD stages, the mRNA and protein levels of ELA were determined at different time points after uI/R injury. Consistent with our previous study, *Apela* level was downregulated at day one and continued decreasing until day seven after the injury (AKI stage) and normalized at day 21 after the injury (AKI-CKD stage; Figure S2A). Immunofluorescence staining suggested that ELA was predominantly expressed in renal tubules, including proximal tubules and distal tubules (as demonstrated by co-staining with LTL and PNA, respectively), with very weak glomerular staining under non-injury conditions (Figure S2B-D). Consistent with transcriptional changes, the protein level of ELA was decreased in proximal and distal tubules from day one to day seven after the injury, and back to normal at day 21 after the injury (Figure S2B-C).

### *Apela^Ksp^* KO mice show aggravated AKI and exacerbated AKI-CKD transition

**Figure 1:**
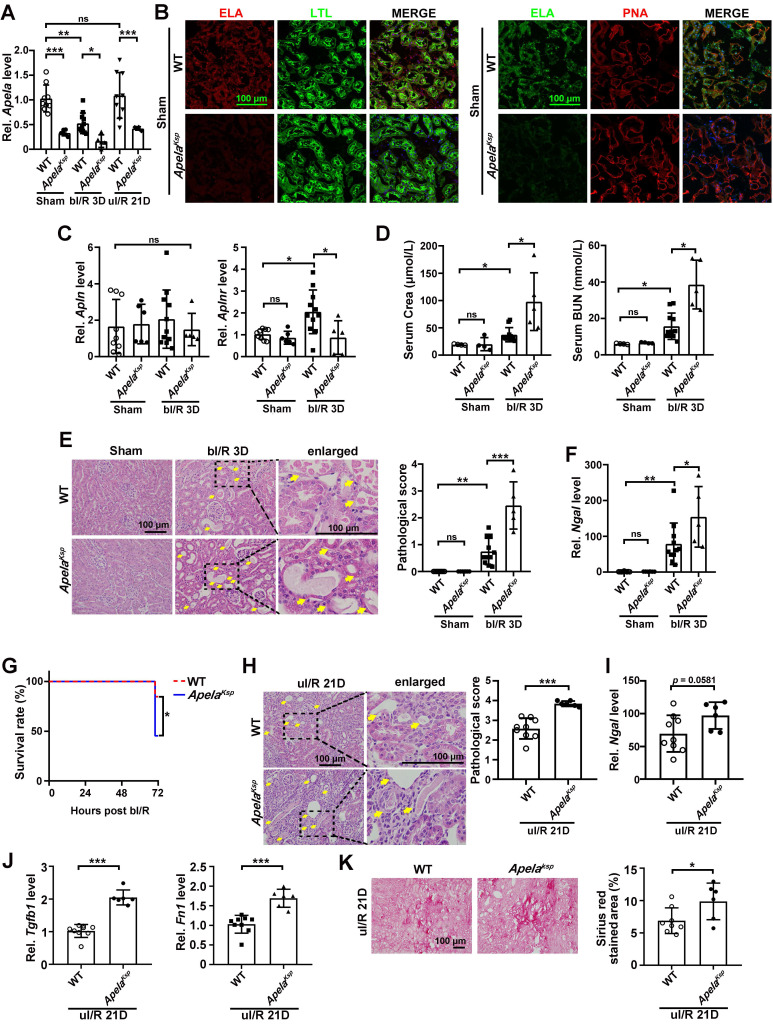
** Tubule-specific ablation of ELA aggravates the I/R-induced renal injury. (A)** qPCR results of *Apela* for WT and *Apela^Ksp^* KO mice at sham-operated non-injured conditions (Sham) or at 3 days after bilateral I/R injury (bI/R 3D) or at 21 days after unilateral I/R injury (uI/R 21D). **(B)** Representative images of co-staining for ELA with LTL (lotus tetragonolobus lectin, marker for proximal tubules; green; left) or PNA (peanut agglutinin, marker for distal tubules; red; right) of sham-operated (Sham) non-injured WT and *Apela^Ksp^* KO mice. DAPI (blue) stained nuclei. **(C)** qPCR results of *Apln* and *Aplnr* for WT and *Apela^Ksp^* KO mice at Sham or at bI/R 3D. **(D)** Serum creatinine and BUN (blood urea nitrogen) levels for WT and *Apela^Ksp^* KO mice for indicated groups. **(E)** Representative H&E images (left) with injury scores (right) at Sham or at bI/R 3D. Yellow arrows indicate injured tubules. **(F)** qPCR results of *Ngal* for WT and *Apela^Ksp^* KO mice at Sham or at bI/R 3D. **(G)** Survival rate of WT (n = 13) and *Apela^Ksp^* KO mice (n = 11) were subjected to bI/R (*P < 0.05; Log-rank Mantel-Cox test). **(H)** Representative H&E images (left) with injury scores (right) for WT and *Apela^Ksp^* KO mice at uI/R 21D.Yellow arrows indicate injured tubules. **(I)** qPCR results of *Ngal* for WT and *Apela^Ksp^* KO mice at uI/R 21D. **(J)** qPCR results of *Tgfb1* (left) and *Fn1* (right) for WT and *Apela^Ksp^* KO mice at uI/R 21D. **(K)** Representative images of Sirius Red staining (right) with quantitative results (left) for WT and *Apela^Ksp^* KO mice at uI/R 21D. Scale bar = 100 μm; Except panel G, WT Sham, n = 9; *Apela^Ksp^* KO Sham, n = 6; WT bI/R 3D, n = 11; *Apela^Ksp^* KO bI/R 3D, n = 5; WT I/R u21D, n = 9; *Apela^Ksp^* KO uI/R 21D, n = 6; *P < 0.05; **P < 0.01; ***P < 0.001; ns, not significant.

Since renal ELA is mostly expressed in tubules (Figure S2B-C), we generated renal tubular specific *Apela* knockout (*Apela^Ksp^* KO) mice. As expected, under normal conditions and at day three after bilateral renal I/R injury (bI/R 3D), ELA mRNA and protein levels were reduced in the kidneys of *Apela^Ksp^* KO mice compared with those of the WT mice (Figure 1A-B & Figure S3). Meanwhile, similar *Apln* (encoding apelin, another endogenous ligand for APJ) level was found in the kidneys of *Apela^Ksp^* KO and WT mice with or without injury (Figure 1C); increased *Aplnr* (encoding APJ) level was found in injured kidneys of WT mice, which was downregulated in injured kidneys of *Apela^Ksp^* KO mice (Figure 1C). However, similar protein level of APJ was found between WT and *Apela^Ksp^* KO mice with or without injury (Figure S4). Renal APJ was mostly expressed in proximal tubules and glomeruli (Figure S4), as previously described 31.

Serum creatinine and BUN levels were significantly increased in *Apela^Ksp^* KO mice at bI/R 3D, indicating a further reduced renal function in these mice (Figure 1D). Consistently, *Apela^Ksp^* KO mice exhibited more severe tubular injury (characterized by tubular cell depletion, tubular dilation, cast formation in the tubular lumens, loss of brush border), and significantly higher tubular injury scores at bI/R 3D (Figure 1E). Meanwhile, the transcriptional level of the kidney injury marker* Ngal* 32 was also increased in the injured kidneys of *Apela^Ksp^* KO mice compared with those of WT mice (Figure 1F). Moreover, the survival rate of *Apela^Ksp^* KO mice was significantly reduced at bI/R 3D (Figure 1G). There was no significant difference in renal function, pathological score of tubular injury and *Ngal* level between WT and *Apela^Ksp^* KO mice without injury (Figure 1D-F). These data suggest that losing ELA in tubular cells aggravated I/R-induced AKI.

To further assess the AKI-CKD transition in *Apela^Ksp^* KO mice, a unilateral I/R model was applied to avoid the high mortality caused by bilateral renal I/R 33. *Apela^Ksp^* KO mice showed more severe tubular injury and increased* Ngal* level compared with those of WT mice at day 21 after the injury (uI/R 21D; Figure 1H-I). Furthermore, *Apela^Ksp^* KO mice showed increased *Tgfb1* and *Fn1* levels as well as increased fibrotic score at uI/R 21D (Figure 1J-K). Thus, losing ELA in tubular cells also aggravates AKI-CKD transition induced by renal I/R.

### Increased inflammation may contribute to enhanced AKI-CKD transition in *Apela^Ksp^* KO mice

**Figure 2:**
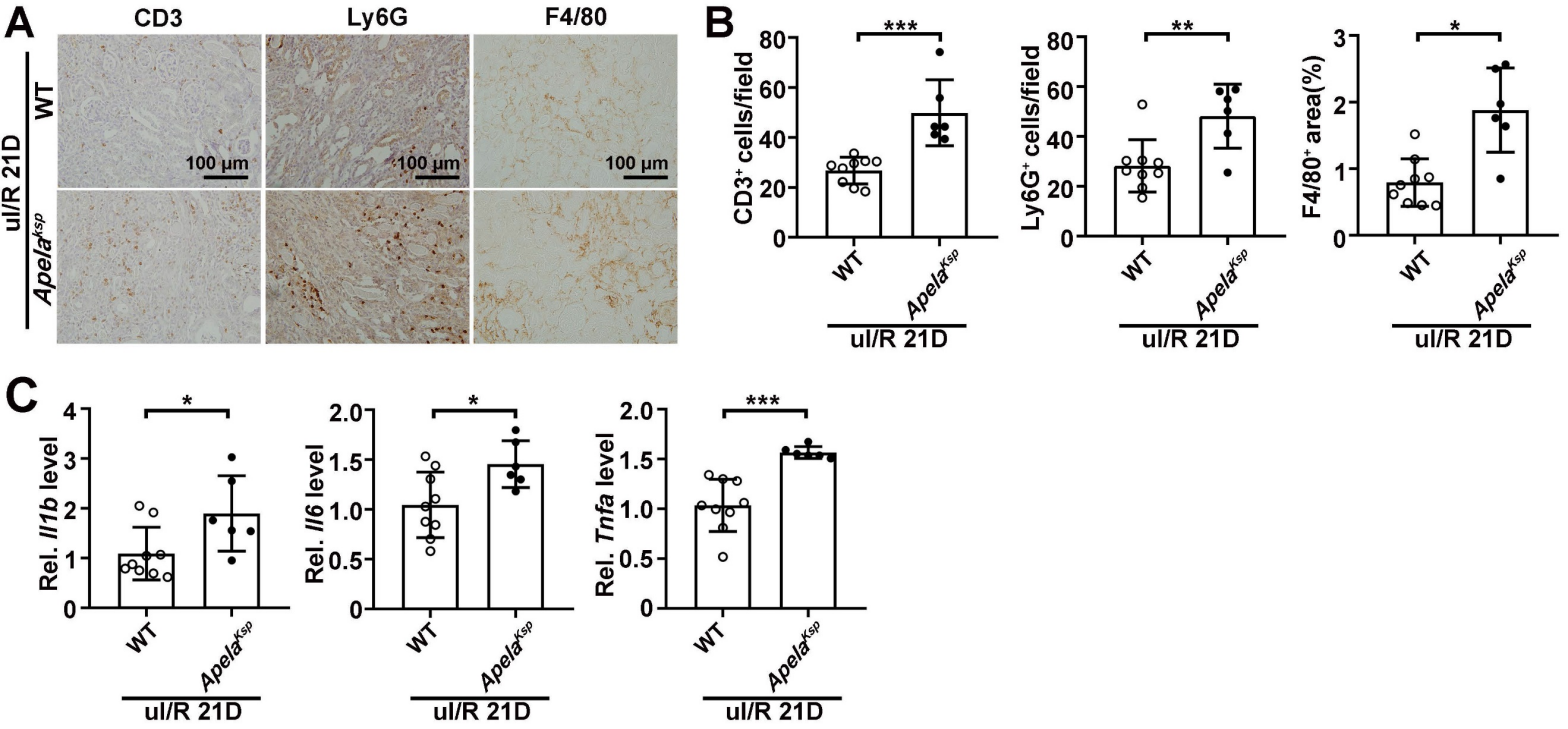
** Increased inflammation contributes to enhanced AKI-CKD transition in *Apela^Ksp^* KO mice. (A-B)** Representative images of CD3, Ly6G and F4/80 (A) with quantitative results (B) for WT and *Apela^Ksp^* KO mice at 21 days after unilateral I/R injury (uI/R 21D). **(C)** qPCR results of *Il1b*, *Il6*, and *Tnfa* for WT and *Apela^Ksp^* KO mice at uI/R 21D. Scale bar = 100 μm; WT uI/R 21D, n = 9; *Apela^Ksp^* KO uI/R 21D, n = 6; *P < 0.05; **P < 0.01; ***P < 0.001.

Since exacerbated inflammation fosters the development of renal I/R 34, we further analyzed whether increased inflammation contributes to the aggravated AKI and AKI-CKD transition observed in *Apela^Ksp^* KO mice. In the sham-operated non-injured conditions, there was no significant difference in transcriptional levels of inflammatory genes in WT and *Apela^Ksp^* KO mice (Figure S5A). In the AKI-CKD transition stage (uI/R 21D), the numbers of infiltrated macrophages (F4/80^+^ cells), neutrophils (Ly6G^+^ cells) and lymphocytes (CD3^+^ cells) were increased in injured kidneys of WT mice, which were further increased in injured *Apela^Ksp^* KO mice (Figure 2A-B). The transcriptional levels of inflammatory genes like *Il1b*, *Il6*, and *Tnfa* were consistently upregulated in injured *Apela^Ksp^* KO mice compared with those of injured WT mice (Figure 2C). Whereas during the AKI stage (bI/R 3D), no significant difference in the transcriptional levels of inflammatory genes, and numbers of infiltrated neutrophils/ lymphocytes was found between the WT and *Apela^Ksp^* KO mice (Figure S5A-B). Together, these results suggest that exacerbated inflammation may contribute to the aggravated injury observed in *Apela^Ksp^* KO mice at the AKI-CKD transition stage, but not at AKI stage.

### ELA negatively affects renal microvascular flow regulating genes in injured kidneys

To identify altered pathways that may be responsible for aggravated AKI in *Apela^Ksp^* KO mice, the renal global gene expression profile was examined in an unbiased study using RNA-sequencing at bI/R 3D. Compared with injured WT mice, tubule-specific ablation of *Apela* led to 889 differentially expressed genes under similar injury, among which *Arg2* (encoding arginase 2), *Cbr1* and *Cbr3* (encoding carbonyl reductase 1 and 3, respectively) were highly upregulated (Figure S6 and Table S3).

**Figure 3:**
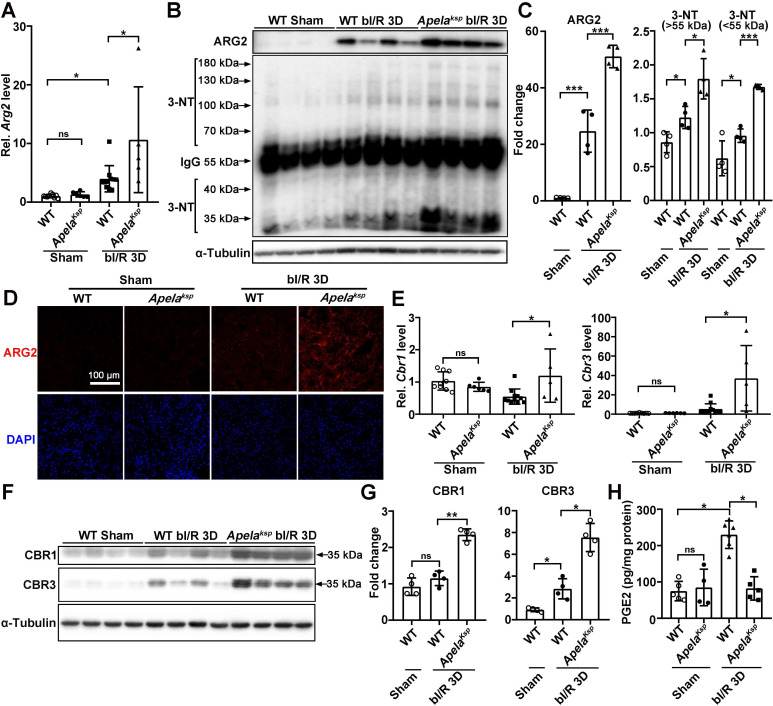
** Tubule-specific ablation of ELA upregulates ARG2 and CBR1/CBR3 levels at AKI stage. (A)** qPCR results of *Arg2* for WT and *Apela^Ksp^* KO mice at sham-operated non-injured conditions (Sham) or at 3 days after bilateral I/R injury (bI/R 3D). **(B-C)** Immunoblots (B) with quantitative results (C) of ARG2 and 3-NT in indicated experimental groups. **(D)** Representative immunofluorescent staining for ARG2 for WT and *Apela^Ksp^* KO mice at Sham or at bI/R 3D. **(E)** qPCR results of *Cbr1* and* Cbr3* for WT and *Apela^Ksp^* KO mice at Sham or at bI/R 3D. **(F-G)** Immunoblots (F) with quantitative results (G) of CBR1 and CBR3 for indicated experimental groups. **(H)** PGE2 ELISA results for indicated experimental groups. Scale bar = 100 μm; WT Sham, n = 9; *Apela^Ksp^* KO Sham, n = 6; WT bI/R 3D, n = 11; *Apela^Ksp^* KO bI/R b3D, n = 5; *P < 0.05; **P < 0.01; ***P < 0.001.

At bI/R 3D, the transcription level of *Arg2* was significantly increased in injured kidneys of WT mice, which was further increased in injured kidneys of *Apela^Ksp^* KO mice; while similar *Arg2* level was found between WT and *Apela^Ksp^* KO mice without injury (Figure 3A). ARG2 has been suggested as a mediator of renal I/R through regulation of nitrosative stress 18. Consequently, we examined the levels of ARG2 and 3-nitrotyrosine (3-NT), a nitrosative stress marker 35. At bI/R 3D, elevated ARG2 and 3-NT levels were observed in injured kidneys of WT mice which were further elevated in *Apela^Ksp^* KO mice (Figure 3B-C). Consistently, immunofluorescence study demonstrated that bI/R-induced upregulation of ARG2 (mainly in the renal tubules) in WT mice was further upregulated in injured renal tubules of *Apela^Ksp^* KO mice; while similar ARG2 level was found between sham-operated WT and *Apela^Ksp^* KO mice (Figure 3D).

Carbonyl reductase 1 (CBR1) and 3 (CBR3) are iso-enzymes involved in PGE2 metabolism, which is the most abundant renal prostaglandin and plays important physiological and pathological roles in the kidney 36-38. At bI/R 3D, greater upregulation of CBR1/CBR3 mRNA and protein levels, as well as consistently reduced renal PGE 2 level, were found in the kidneys of *Apela^Ksp^* KO mice compared with those of WT mice (Figure 3E-H). Increased renal ARG2 has been suggested to impair microvascular function 39, 40, while PGE2 directly increases renal blood flow and maintains microvascular blood flow 41, 42. Therefore, the exacerbated injury in *Apela^Ksp^* KO mice after I/R may result from increased disruption of renal microvascular flow due to the altered ARG2 and CBR1/3 levels.

### Impaired renal microvasculature contributes to the aggravated AKI and AKI-CKD transition in *Apela^Ksp^* KO mice

**Figure 4:**
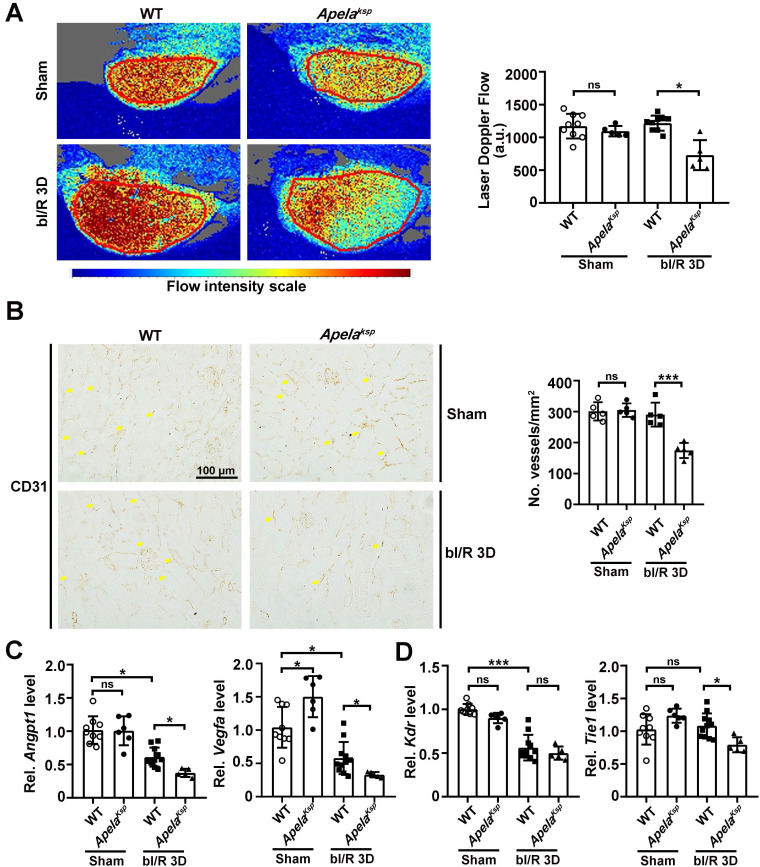
** Tubule-specific ablation of ELA aggravates renal microvascular injury at AKI stage. (A)** Representative images (left) with quantitative results (right) of renal microcirculation for WT and *Apela^Ksp^* KO mice at sham-operated non-injured conditions (Sham) or at 3 days after bilateral I/R injury (bI/R 3D). WT Sham, n = 9; *Apela^Ksp^* KO Sham, n = 6; WT bI/R 3D, n = 11; *Apela^Ksp^* KO bI/R 3D, n = 5. **(B)** Representative images (left) with quantitative results (right) of CD31 staining for WT and *Apela^Ksp^* KO mice at Sham or bI/R 3D. WT Sham, n = 5; *Apela^Ksp^* KO Sham, n = 5; WT bI/R 3D, n = 5; *Apela^Ksp^* KO bI/R 3D, n = 5. **(C-D)** qPCR results of *Angpt1* and* Vegfa* (C) with *Kdr* and* Tie1* (D) for WT and *Apela^Ksp^* KO mice at Sham or bI/R 3D. WT Sham, n = 9; *Apela^Ksp^* KO Sham, n = 6; WT bI/R 3D, n = 11; *Apela^Ksp^* KO bI/R 3D, n = 5. Scale bar = 100 μm; *P < 0.05; **P < 0.01; ***P < 0.001; ns, not significant.

To assess renal microvascular flow, laser speckle contrast analysis was performed. At bI/R 3D, the renal microvascular flow of *Apela^Ksp^* KO mice was significantly reduced compared with that of WT mice (Figure 4A). Moreover, the number of CD31-stained vessels as well as the transcriptional levels of angiogenic factors, including *Angpt1* and *Vegfa*, were significantly downregulated in injured* Apela^Ksp^* KO mice compared to those of injured WT mice (Figure 4B-C); while the transcription level of the Angpt1 receptor* Tie1*, but not the Vegfα receptor* Kdr*, was also significantly downregulated in injured* Apela^Ksp^* KO mice (Figure 4D). There was no significant difference in microvascular flow, CD31-staining, transcription levels of angiogenic factors and their receptors between non-injured WT and *Apela^Ksp^* KO mice (Figure 4).

To determine whether the reduced blood flow, in addition to elevated inflammation and fibrosis, contributes to the exacerbated AKI-CKD transition in* Apela^Ksp^* KO mice, renal microvascular flow was measured at uI/R 21D. Compared to injured WT mice, microvascular flow was significantly reduced, together with fewer CD31-stained vessels in injured kidneys of *Apela^Ksp^* KO mice (Figure S7A-B). Furthermore, the transcriptional levels of angiogenic factors and their receptors were also significantly reduced in injured *Apela^Ksp^* KO mice (Figure S7C). These results indicate that exacerbated renal vascular injury, and the consequent vascular dysfunction, may aggravate both the AKI and AKI-CKD transition in *Apela^Ksp^* KO mice.

### Arginase inhibitor and PGE2 activators attenuate AKI-induced injury in C57BL/6 mice

**Figure 5:**
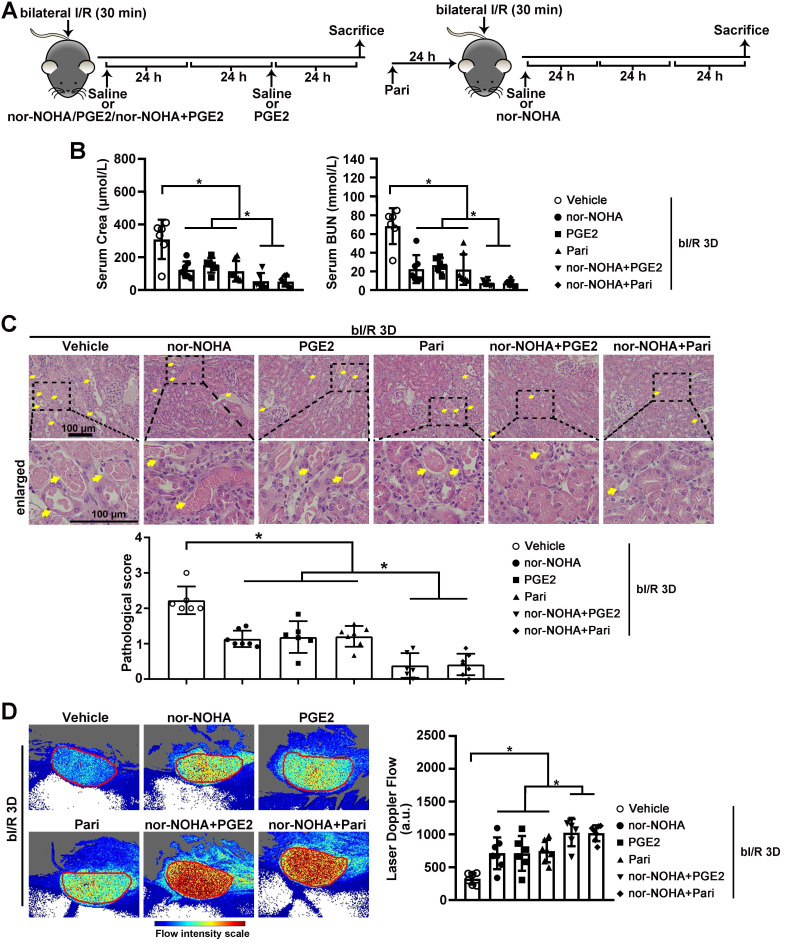
** Combined administration of arginase inhibitor and PGE2 activators efficiently attenuates AKI-induced injury. (A)** Experimental design for nor-NOHA, PGE2 and Paricalcitol treatments on the AKI stage of renal I/R injury in WT mice. **(B)** Serum creatinine (left) and BUN (right) levels for indicated experimental groups at 3 days after bilateral I/R injury (bI/R 3D). **(C)** Representative H&E images (left) with injury scores (right) for different experimental groups at bI/R 3D. Yellow arrows indicate injured tubules.** (D)** Representative images (left) with quantitative results (right) of renal microcirculation for different experimental groups at bI/R 3D. Scale bar = 100 μm; bI/R 3D Vehicle, mice underwent bI/R injury treated with vehicle for 3 days, n = 6; bI/R 3D nor-NOHA, mice underwent bI/R injury treated with nor-NOHA for 3 days, n = 7; bI/R 3D PGE2, mice underwent bI/R injury treated with PGE2 for 3 days, n = 6; bI/R 3D Pari, mice underwent bI/R injury treated with Paricalcitol for 3 days, n = 7; bI/R 3D nor-NOHA+PGE2, mice underwent bI/R injury treated with nor-NOHA plus PGE2 for 3 days, n = 6; bI/R 3D nor-NOHA+Pari, mice underwent bI/R injury treated with nor-NOHA plus Paricalcitol for 3 days, n = 7; *P < 0.05; **P < 0.01; ***P < 0.001.

Next, we investigated whether normalizing arginase activity and/or PGE2 level can restore renal microvascular flow and alleviate AKI-induced injury. Two FDA approved medications, PGE2 and a PGE2 synthesis activator Paricalcitol, as well as the arginase inhibitor nor-NOHA, were administrated individually or in combination to injured C57BL/6 mice (Figure 5A). At bI/R 3D, serum creatinine and BUN levels were significantly reduced in injured C57BL/6 mice treated with the individual drug, and were further reduced by combined treatments of nor-NOHA plus either PGE2 or Paricalcitol (Figure 5B). Consistently, AKI-induced tubular injury was significantly attenuated in C57BL/6 mice treated with the individual drug and was further reduced by combined treatments (Figure 5C). Furthermore, renal microvascular flow was significantly improved in injured C57BL/6 mice treated by the individual drug, and was further improved by combined treatments (Figure 5D).

### Combined administration of nor-NOHA and Paricalcitol rescued the worse AKI phenotype in *Apela^Ksp^* KO mice

**Figure 6:**
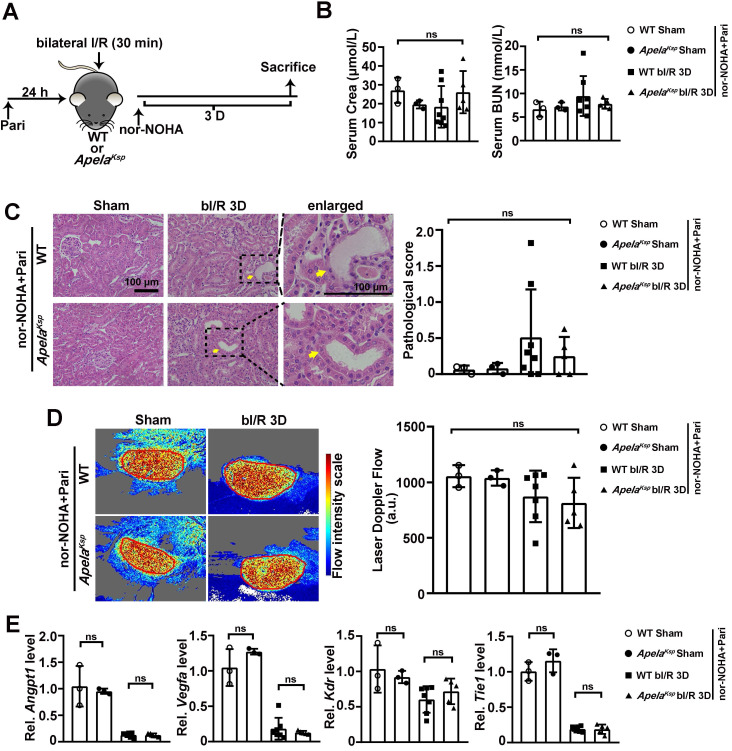
** Combined administration of nor-NOHA and Paricalcitol normalizes the worse AKI phenotype in *Apela^Ksp^* KO mice. (A)** Experimental design for nor-NOHA and Paricalcitol treatments on the AKI stage of renal I/R injury in *Apela^Ksp^* KO mice. **(B)** Serum creatinine (left) and BUN (right) levels for WT and *Apela^Ksp^* KO mice under combined administration of nor-NOHA and Paricalcitol at sham-operated non-injured conditions (Sham) or at 3 days after bilateral I/R injury (bI/R 3D). **(C)** Representative H&E images (left) with injury scores (right) for WT and *Apela^Ksp^* KO mice under combined administration of nor-NOHA and Paricalcitol at Sham or bI/R 3D. Yellow arrows indicate injured tubules. **(D)** Representative images (left) with quantitative results (right) of renal microcirculation for WT and *Apela^Ksp^* KO mice under combined administration of nor-NOHA and Paricalcitol at Sham or bI/R 3D. **(E)** qPCR results of *Angpt1*,* Vegfa*, *Kdr* and* Tie1* for WT and *Apela^Ksp^* KO mice received combined administration of nor-NOHA and Paricalcitol at Sham or bI/R 3D. Scale bar = 100 μm; WT Sham nor-NOHA+Pari, WT mice underwent sham-operated treated with nor-NOHA plus Paricalcitol, n = 3; *Apela^Ksp^* KO Sham nor-NOHA+Pari, *Apela^Ksp^* KO mice underwent sham-operated treated with nor-NOHA plus Paricalcitol, n = 3; WT bI/R 3D nor-NOHA+Pari, WT mice underwent bI/R injury treated with nor-NOHA plus Paricalcitol for 3 days, n = 8; *Apela^Ksp^* KO bI/R 3D nor-NOHA+Pari, *Apela^Ksp^* KO mice underwent bI/R injury treated with nor-NOHA plus Paricalcitol for 3 days, n = 5; ns, not significant.

We further investigated whether nor-NOHA and Paricalcitol combined treatment can reverse the severe AKI observed in *Apela^Ksp^* KO mice (Figure 6A). At bI/R 3D, similar serum creatinine and BUN levels were found between the WT and *Apela^Ksp^* KO mice under the combined treatment (Figure 6B). No significant difference in tubular injury or renal microvascular flow was found between injured *Apela^Ksp^* KO and WT mice treated with nor-NOHA and Paricalcitol at bIRI 3D (Figure 6C-D). Moreover, similar transcriptional levels of angiogenic factors,* Angpt1* and *Vegfa*, and Angpt1 receptor* Tie1* were found in injured *Apela^Ksp^* KO mice and WT mice treated with nor-NOHA and Paricalcitol (Figure 6E). Meantime, combined treatments did not affect renal function, pathological score, renal microvascular flow, transcriptional levels of angiogenic factors and their receptors in non-injured WT and *Apela^Ksp^* KO mice (Figure 6B-E). Together, these results suggest a synergistic beneficial effect of an arginase inhibitor and a PGE2 synthesis activator on aggravated AKI in* Apela^Ksp^* KO mice.

### Combined administration of nor-NOHA and Paricalcitol attenuated AKI-CKD transition

**Figure 7:**
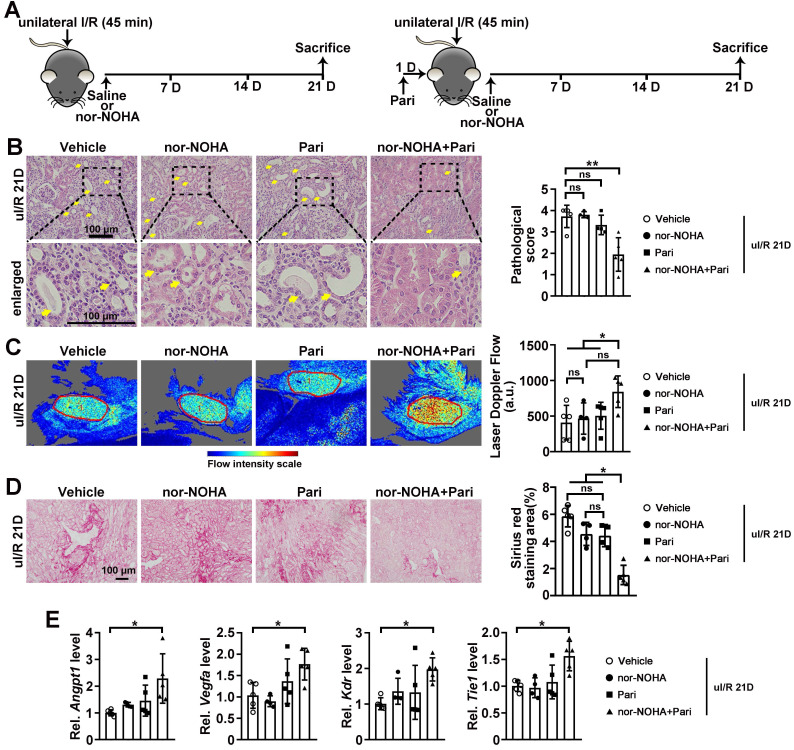
** Combined administration of nor-NOHA and Paricalcitol attenuates AKI-CKD transition. (A)** Experimental design for nor-NOHA and Paricalcitol treatments on long-term renal I/R injury (at 21 days after unilateral I/R injury, uI/R 21D). **(B)** Representative H&E images (left) with injury scores (right) for indicated experimental groups at uI/R 21D. Yellow arrows indicate injured tubules. **(C)** Representative images (left) with quantitative results (right) of renal microcirculation for indicated experimental groups at uI/R 21D. **(D)** Representative images of Sirius Red staining (right) with quantitative results (left) for different experimental groups at uI/R 21D. **(E)** qPCR results of *Angpt1*,* Vegfa*, *Kdr* and* Tie1* for indicated experimental groups at uI/R 21D. Scale bar = 100 μm; uI/R 21D Vehicle, mice underwent uI/R injury treated with vehicle for 21 days, n = 5; uI/R 21D nor-NOHA, mice underwent uI/R injury treated with nor-NOHA for 21 days, n = 4; uI/R 21D Pari, mice underwent uI/R injury treated with Paricalcitol for 21 days, n = 4; uI/R 21D nor-NOHA+Pari, mice underwent uI/R injury treated with nor-NOHA plus Paricalcitol for 21 days, n = 5; *P < 0.05; **P < 0.01; ns, not significant.

Further, we determined whether drugs that normalize arginase activity and/or PGE2 level can attenuate AKI-CKD transition by restoring renal microvascular flow (Figure 7A). At uI/R 21D, tubular injury was significantly attenuated when injured mice were treated with combined nor-NOHA and Paricalcitol, but not by individual treatment (Figure 7B). Consistently, improved renal microvascular flow and reduced renal fibrotic score were only found when injured mice received the combined nor-NOHA and Paricalcitol treatment, but not by individual treatment (Figure 7C-D). Significantly upregulated transcriptional levels of *Angpt1*, *Vegfa*, *Kdr* and *Tie1*, were found in the kidneys of injured mice after the combined treatment (Figure 7E).

### ELA regulated renal microvascular flow through APJ

**Figure 8:**
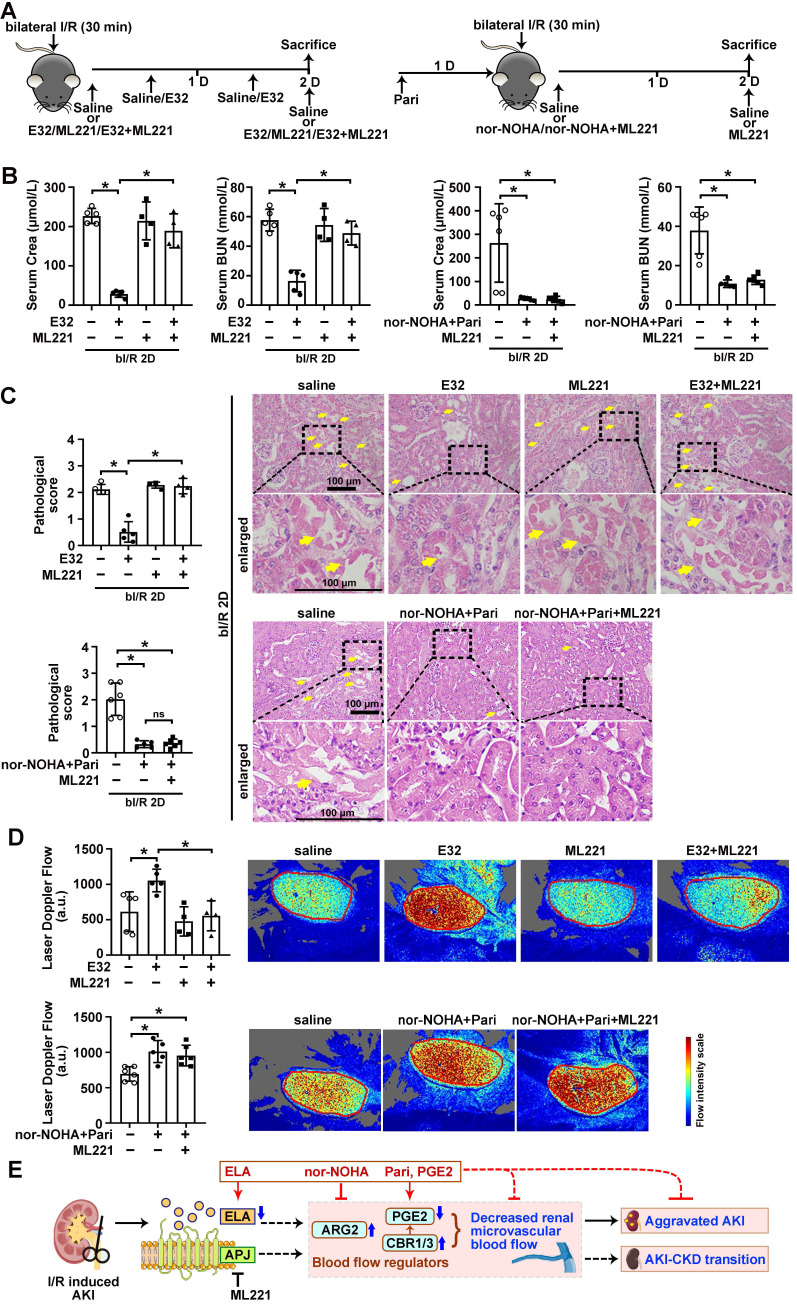
** ELA regulates renal microvascular flow through APJ. (A)** Experimental design for E32, ML221, nor-NOHA and Paricalcitol treatments on the AKI stage of renal I/R injury. **(B)** Serum creatinine (left) and BUN (right) levels for indicated experimental groups at 2 days after bilateral I/R injury (bI/R 2D). **(C)** Representative H&E images (right) with injury scores (left) for indicated experimental groups at bI/R 2D. Yellow arrows indicate injured tubules. **(D)** Representative images (right) with quantitative results (left) of renal microcirculation for indicated experimental groups at bI/R 2D. **(E)** A schematic model of the ELA-APJ axis mediated pathways involved in AKI and potential therapeutic interventions. Scale bar = 100 μm; bI/R 2D Saline, mice underwent bI/R injury treated with saline for 2 days, n = 11; bI/R 2D E32, mice underwent bI/R injury treated with E32 for 2 days, n = 5; bI/R 2D ML221, mice underwent bI/R injury treated with ML221 for 2 days, n = 4; bI/R 2D E32+ML221, mice underwent bI/R injury treated with E32 plus ML221 for 2 days, n = 4; bI/R 2D nor-NOHA+Pari, n = 5; I/R 2D nor-NOHA+Pari+ML221, mice underwent bI/R injury treated with nor-NOHA plus Paricalcitol and ML221 for 2 days, n = 6; *P < 0.05; ns, not significant.

Finally, to investigate whether the regulation of renal microvascular flow by ELA depends on APJ, an APJ inhibitor ML221 was used (Figure 8A). Under bI/R injury, significant reduction of serum creatinine and BUN levels were achieved by ELA32 treatment or combined treatment of nor-NOHA plus Paricalcitol, however, the beneficial effect of ELA32 peptide on renal function was completely abolished by the ML221; while the beneficial effect on renal function by combined treatment was not affected by the ML221 (Figure 8B). Similarly, AKI-induced tubular injury and reduction of renal microvascular flow were also significantly attenuated in mice treated with ELA32 peptide, which were abolished by ML221; while the beneficial effects by combined treatment of nor-NOHA plus Paricalcitol on tubular injury and renal microvascular flow on injured mice were not affected by ML221 (Figure 8C-D). These results suggest that ELA protects against AKI partly through regulating renal microvascular flow in an APJ dependent mechanism.

## Discussion

AKI is a global health problem. Despite the research and clinical advances into supportive measures and prophylactic approaches, the mortality rate of AKI patients remains high 43. Here, we found that endogenous tubular ELA played critical beneficial roles during I/R-induced AKI and the following AKI-CKD transition stages. Tubule-specific ablation of ELA led to alteration of several factors that affect renal microvascular flow, such as ARG2 and PGE2 metabolizing enzyme CBR1/3, during renal I/R (Figure 3). ARG2 is involved in nitric oxide homeostasis and the development of vascular disease 44, 45; while the competitive arginase inhibitor, nor-NOHA, reduces its activity 46. Moreover, PGE2 and Paricalcitol, both FDA approved medications, not only increases renal blood flow (Figures 5 & 7), but also show antioxidant, anti-inflammatory, and antiapoptotic properties upon renal I/R injury *via* activating E-prostanoid 4 receptor (EP4) pathway 19, 47. Importantly, combined administration of nor-NOHA and Paricalcitol effectively attenuated tubular and microvascular injury after renal I/R in both AKI and AKI-CKD transition stages (Figures 5 & 7). These results demonstrate an important role for endogenous tubular ELA in renal microvasculature homeostasis.

We have previously reported that ELA may exert function through either APJ dependent or independent mechanisms 12. To identify the mechanism that accounts for ELA regulation of renal microvascular flow, an APJ inhibitor ML221 was used. The results clearly suggest that APJ is required for the beneficial effect of ELA on microvascular flow, and demonstrated that the ELA-APJ axis is a novel up-stream regulator of ARG2 and CBR1/3 pathways (Figure 8E). Although previous studies have reported that transcriptional factors such as LXR or NRF2 regulate ARG2 or CBR1 level in immune cells or in the liver 48, 49, regulation of ARG2 or CBRs through cell signaling by a GPCR in the kidneys has not been reported. Therefore, it will be of great interest to explore in detail the regulatory mechanism of ELA-APJ axis on the ARG2 and CBR1/3 pathways.

Reduction of renal blood flow is considered to be one of important pathogenic steps in the development of AKI, which usually results in decreased kidney perfusion. If reduced renal blood flow is reversed rapidly, the kidney is less prone to injury, including structural (acute tubular necrosis) and functional (proteinuria) damages 50-52. Here, we report that the renal tubule specific knockout of ELA reduces renal blood flow and aggravates tubular injury (Figure 4) under the injury, while administration of ELA peptide increases renal blood flow and relieves tubular damage (Figure 8). These results suggest that crosstalk between tubules and blood vessels may involve in AKI and the following AKI-CKD stages.

Administration of either nor-NOHA or Paricalcitol alone as well as in combination alleviated I/R-induced AKI (Figure 5), however, only in combination showed beneficial effects on the AKI-CKD transition (Figure 7). These results not only indicate the complexity of AKI-CKD progression, but more importantly, identify a more effective cocktail therapy against the AKI-CKD progression that warrants future investigation, mechanistically and clinically, especially since Paricalcitol and PGE2 are already FDA-approved medications.

Moreover, our results indicate that attenuated renal blood flow may be one of the key factors in the progression of AKI, and importantly, also in the transition from AKI to CKD. In agreement with our results, a recent study suggested that chronic renal hypoxia caused by poor renal hemodynamic response after AKI may responsible for the progression from AKI to CKD 53. Therefore, we propose that hemodynamic stabilization may be an important therapeutic strategy for treating AKI and the preventing the following AKI-CKD transition. In addition, measurement of renal microvascular flow by non-invasive techniques such as contrast-enhanced ultrasound 54, may provide a good clinical indicator for the progression of AKI and the following transition to CKD.

In summary, we find that the ELA-APJ axis protects against renal I/R by increasing renal microvascular flow *via* regulating ARG2 and CBR1/3. Our results suggest that hemodynamic stabilization may provide a new clinical approach for the assessment, prevention, and treatment of AKI and the following AKI-CKD progression.

## Supplementary Material

Supplementary figures and tables.

## Abbreviations

I/R: ischemia-reperfusion
AKI: acute kidney injury
CKD: chronic kidney disease
IL-6: interleukin-6
BUN: blood urea nitrogen
PNA: peanut agglutinin
LTL: lotus tetragonolobus lectin
DBA: dolichos biflorus agglutinin
ARG2: arginase
PGE2: prostaglandin E2
CBR1: carbonyl reductases 1
CBR3: carbonyl reductases 3
qPCR: quantitative real-time polymerase chain reaction
WT: wildtype
Tnfα: tumor necrosis factor alpha
