# Supplementary material for: Tubular Elabela-APJ axis attenuates ischemia-reperfusion induced acute kidney injury and the following AKI-CKD transition by protecting renal microcirculation
Source: Theranostics. 2023 Jun 4;13(10):3387–401. doi: 10.7150/thno.84308 (PMC10283061; doi:10.7150/thno.84308)
Supplement: Supplementary file 1 — Supplementary figures and tables. [file thnov13p3387s1.pdf]

Supplementary Materials

for

Tubular Elabela-APJ axis attenuates ischemia-reperfusion induced acute kidney injury and the following AKI-CKD transition by protecting renal microcirculation

Mingrui Xiong[1#], Hong Chen[1#], Yu Fan[2], Muchuan Jin[2], Dong Yang[1], Yuchen Chen[1], Yu Zhang[1], Robert B. Petersen[3], Hua Su[4], Anlin Peng[5], Congyi Wang[6], Ling Zheng[2*] & Kun Huang[1*]

[1]Tongji School of Pharmacy, Tongji Medical College and State Key Laboratory for Diagnosis and Treatment of Severe Zoonotic Infectious Diseases, Huazhong University of Science & Technology, Wuhan, China, 430030

[2]Hubei Key Laboratory of Cell Homeostasis, Frontier Science Center for Immunology and Metabolism, College of Life Sciences, Wuhan University, Wuhan, China, 430072

[3]Foundational Sciences, Central Michigan University College of Medicine, Mt. Pleasant, MI, USA, 48859

[4]Department of Nephrology, Union Hospital, Tongji Medical College, Huazhong University of Science and Technology, Wuhan, China, 430030

[5]Department of Pharmacy, The Third Hospital of Wuhan, Tongren Hospital of Wuhan University, Wuhan, China. 430075

[6]The Center for Biomedical Research, Department of Respiratory and Critical Care Medicine, NHC Key Laboratory of Respiratory Disease, Tongji Hospital, Tongji Medical College, Huazhong University of Science & Technology, Wuhan, China. 430030

Corresponding authors

Ling Zheng, Ph.D.	Kun Huang, Ph.D.
College of Life Sciences	Tongji School of Pharmacy
Wuhan University	Huazhong University of Science & Technology
Wuhan, China 430072	Wuhan, China, 430030
lzheng@whu.edu.cn	kunhuang@hust.edu.cn

This supplementary file has 7 figures and 3 tables.

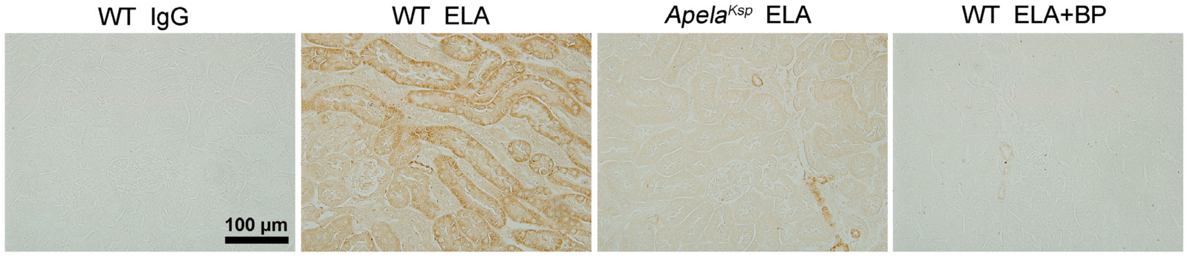

Figure S1. The specificity of the ELA antibody. Representative images of ELA staining (brown). WT IgG: kidney section from WT mouse stained with rabbit IgG; WT ELA: kidney section from WT mouse stained with the ELA antibody (Dia-An Biotech, Wuhan, China); *Apela*^{Ksp} KO ELA: kidney section from *Apela*^{Ksp} KO mouse stained with the ELA antibody; WT ELA+ BP: kidney section from WT mouse stained with the ELA antibody pre-incubated with blocking peptide (BP) at ratio 1:10 for 30 min at room temperature. Scale bar = 100 µm.

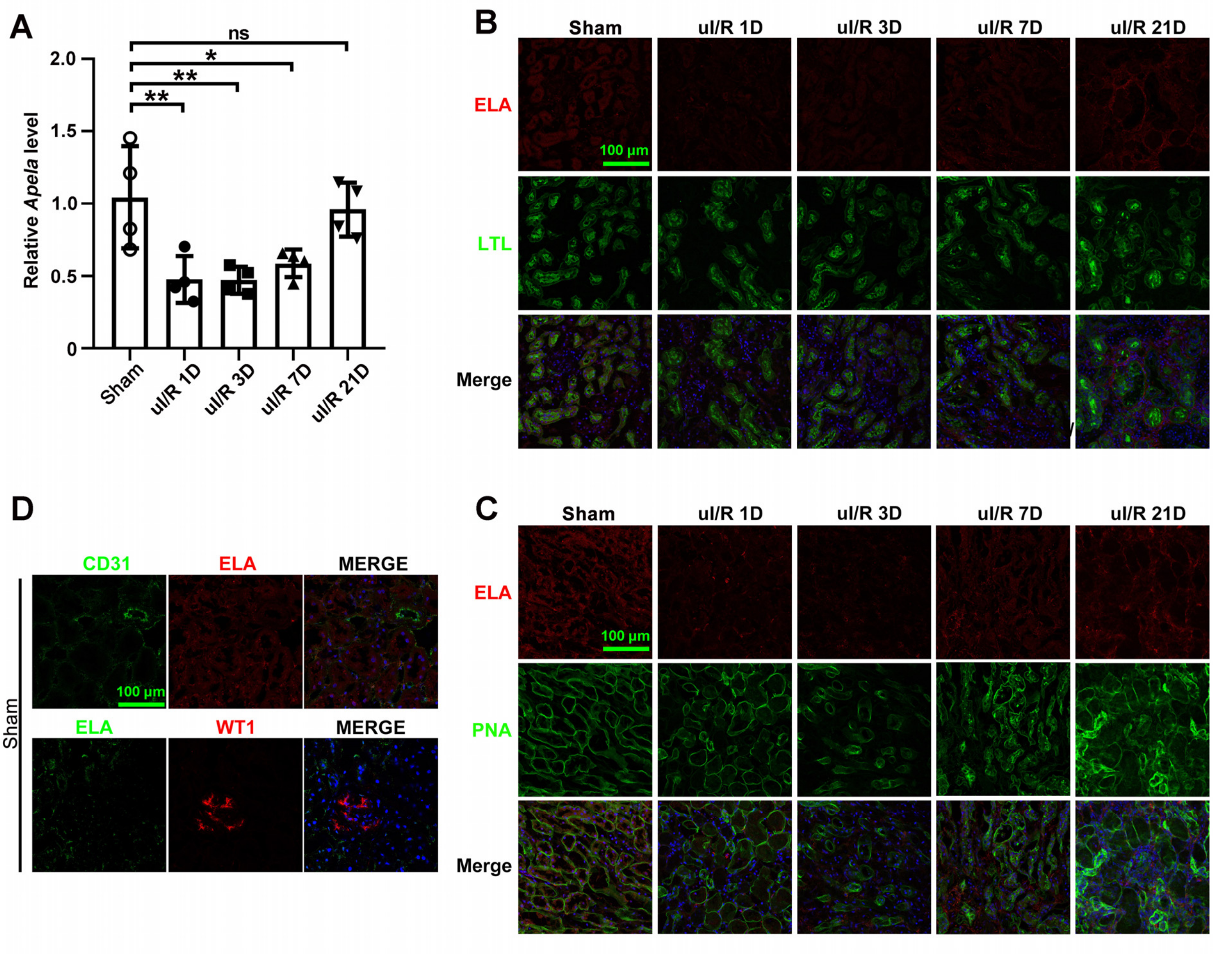

Figure S2. Tubular ELA is downregulated during acute renal uI/R stage. (A) qPCR results of *Apela* of non-injured mice (Sham), or mice at 1, 3, 7, or 21 days after unilateral I/R (uI/R 1D, uI/R 3D, uI/R 7D, or uI/R 21D). **(B-D)** Representative images of co-staining for ELA (red for B, C, D up panel; green, D bottom panel) with LTL (lotus tetragonolobus lectin, marker for proximal tubules; green; B) or PNA (peanut agglutinin, marker for distal tubules; green; C) or CD31 (marker for blood vessel; green; D up panel) or WT1 (marker for glomerulus; red; D bottom panel) of indicated experimental groups. DAPI (blue) stained nuclei; scale bar = 100 μm; Sham, n = 4; uI/R 1D, n = 4; uI/R 3D, n = 4; uI/R 7D, n = 4; uI/R 21D, n = 4; **P* < 0.05, ***P* < 0.01; ns, not significant.

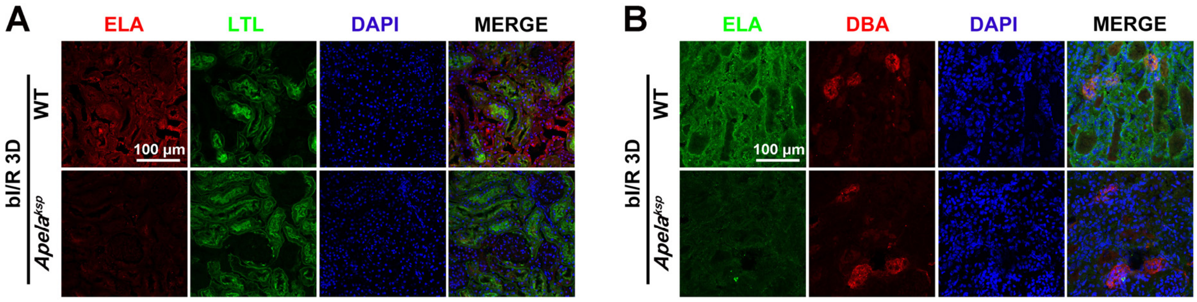

Figure S3. ELA is abolished in the kidneys of *Apela*^{Ksp} KO mice after renal bI/R injury. (A-B) Representative images of co-staining for ELA (red) with LTL (lotus tetragonolobus lectin, a marker for proximal tubules; green) (A) or ELA (green) with DBA (dolichos biflorus agglutinin, a marker for collecting duct; red) (B) of indicated experimental groups. DAPI (blue) stains nuclei; scale bar = 100 µm. bI/R 3D, at 3 days after bilateral I/R injury.

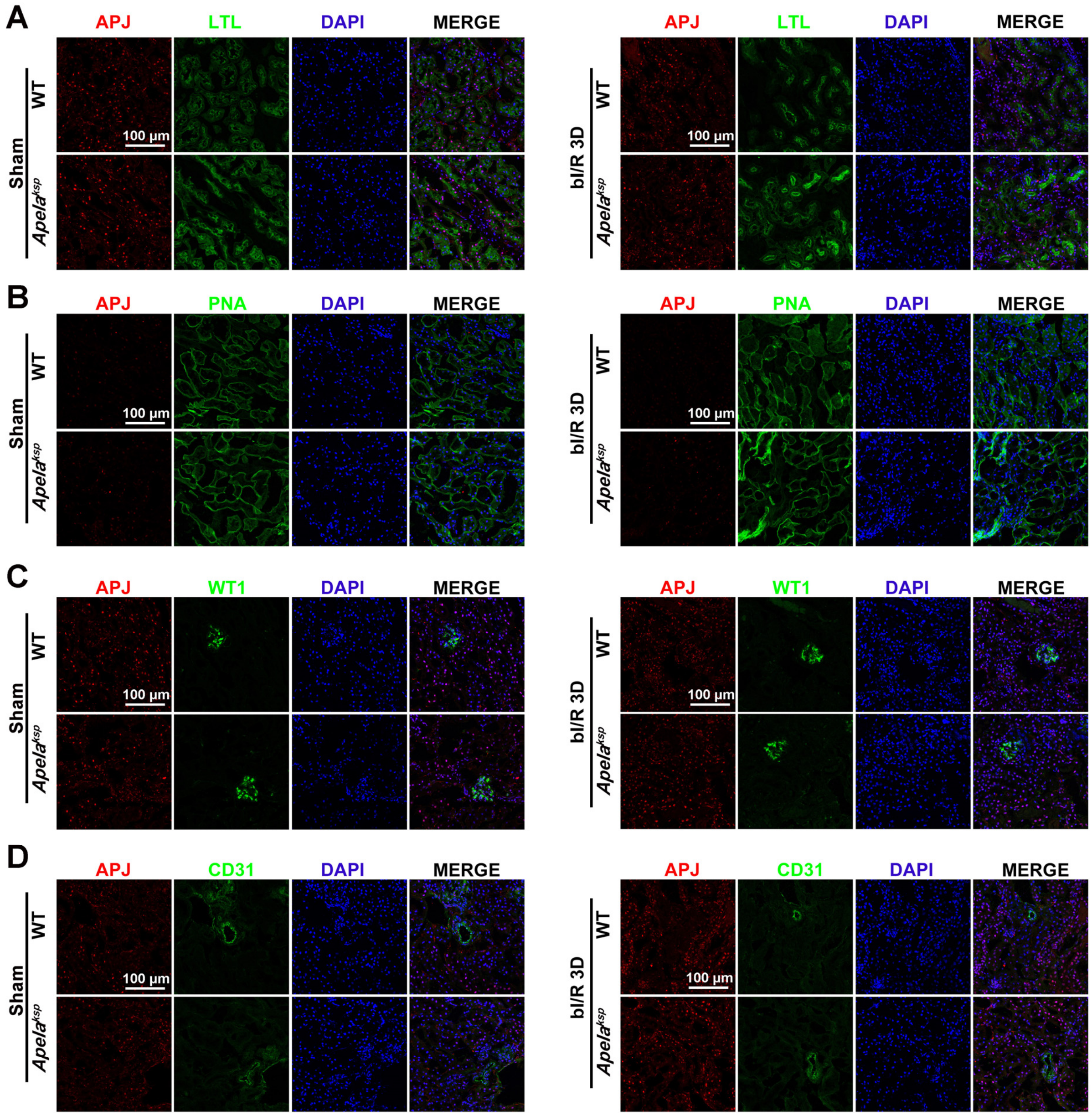

Figure S4. Similar APJ protein level in the kidneys of *Apela*^{Ksp} KO and WT mice. (A-D) Representative images of co-staining for APJ (red) with LTL (lotus tetragonolobus lectin, a marker for proximal tubules; green; A) or with PNA (peanut agglutinin, a marker for distal tubules and collecting duct; green; B) or with WT1 (marker for glomerulus; green; C) or with CD31 (marker for blood vessel; green; D) of indicated experimental groups. DAPI (blue) stains nuclei; scale bar = 100 μm. Sham, sham-operated; bI/R 3D, at 3 days after bilateral I/R injury.

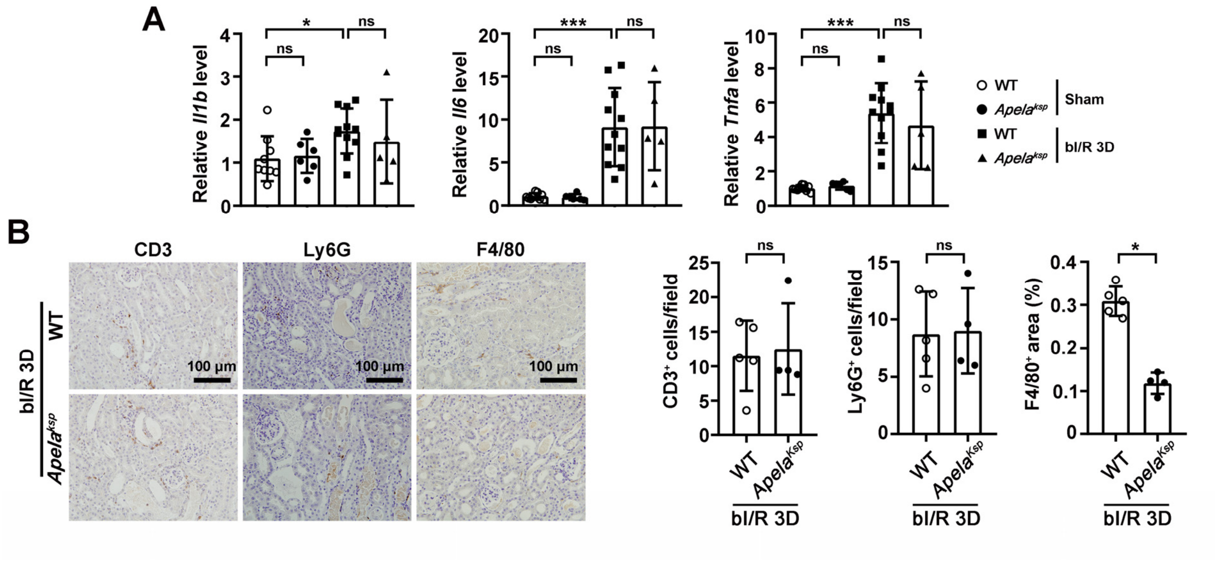

Figure S5. No alteration in renal inflammation of *Apela*^{Ksp} KO mice during the AKI stage. (A) qPCR results of *Il1b*, *Il6*, and *Tnfa* for WT and *Apela*^{Ksp} KO mice at sham-operated (Sham) or at 3 days after bilateral I/R injury (bI/R 3D). WT Sham, n = 9; *Apela*^{Ksp} KO Sham, n = 6; WT bI/R 3D, n = 11; *Apela*^{Ksp} KO bI/R 3D, n = 5. **(B)** Representative images of CD3, Ly6G and F4/80 (left) with quantitative results (right) for WT and *Apela*^{Ksp} KO mice at bI/R 3D. Scale bar = 100 μm; WT bI/R 3D, n = 5; *Apela*^{Ksp} KO bI/R 3D, n = 4; **P* < 0.05; ****P* < 0.001; ns, not significant.

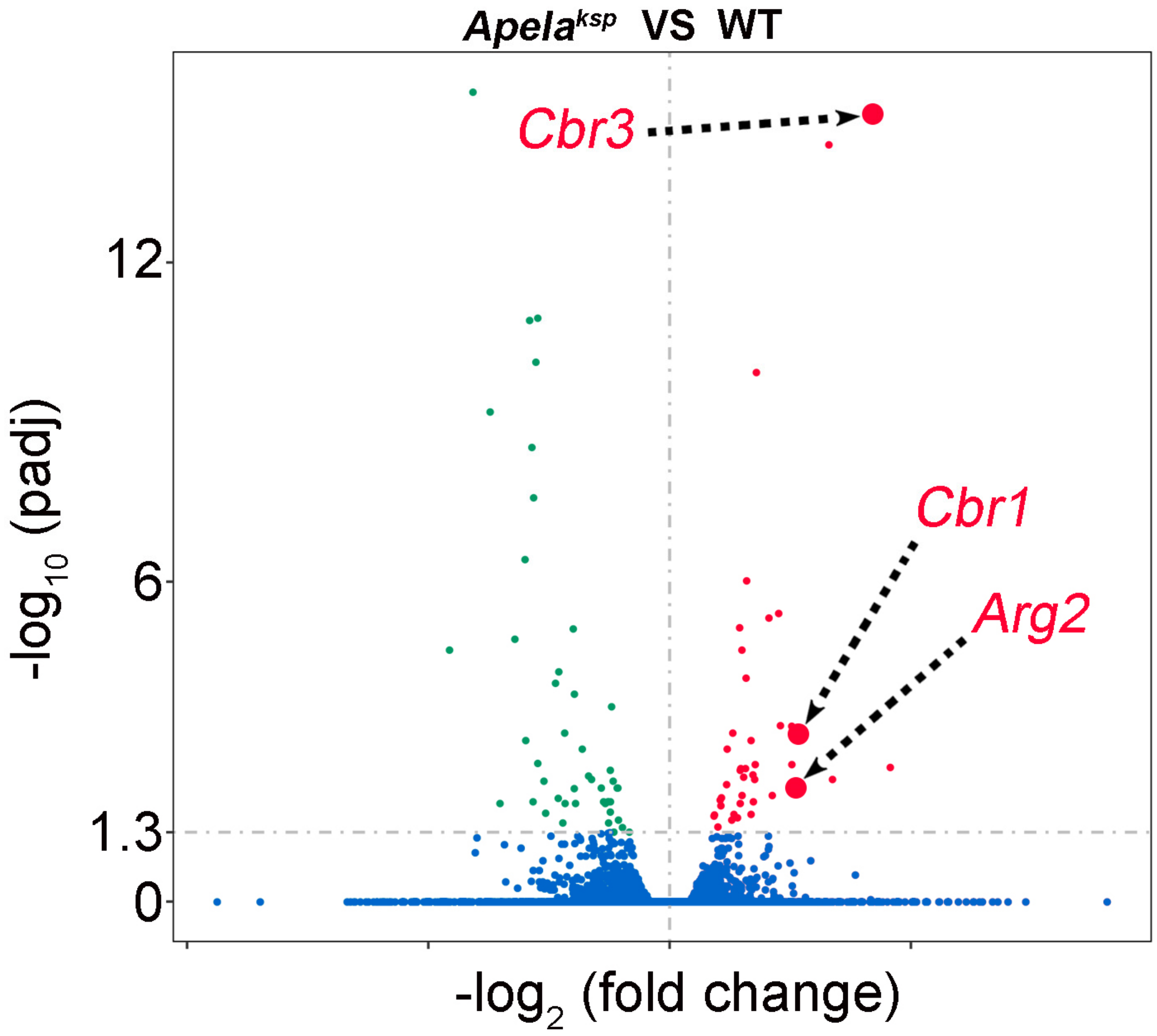

Figure S6. Volcanic map of several highly upregulated genes in injured kidneys of *Apela*^{Ksp} KO mice.

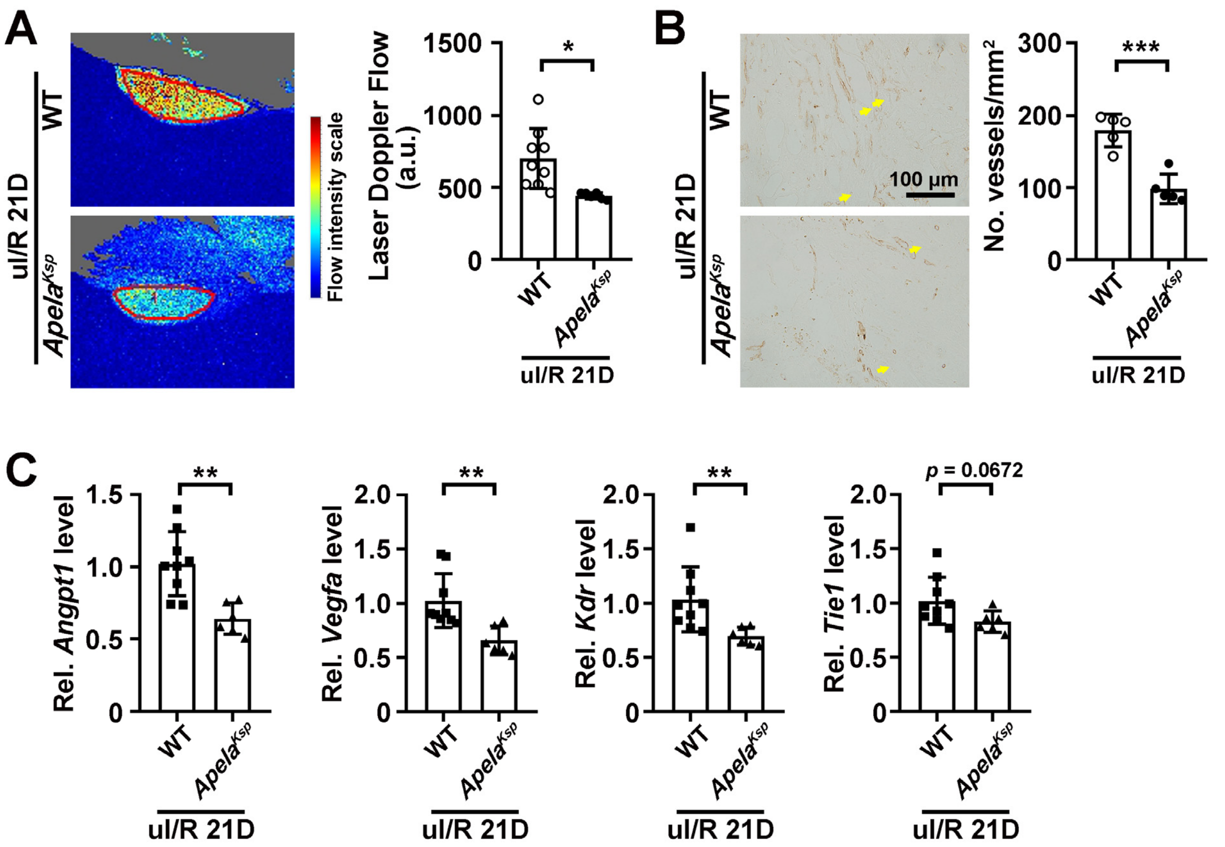

Figure S7. Tubule-specific ablation of ELA aggravates renal microvascular injury at the AKI-CKD transition stage. (A) Representative images (left) with quantitative results (right) of renal microcirculation for WT and *Apela*^{Ksp} KO mice at 21 days after unilateral I/R injury (uI/R 21D). WT uI/R 21D, n = 9; *Apela*^{Ksp} KO uI/R 21D, n = 6. **(B)** Representative images (left) with quantitative results (right) of CD31 staining for WT and *Apela*^{Ksp} KO mice at uI/R 21D. WT uI/R 21D, n = 5; *Apela*^{Ksp} KO uI/R 21D, n = 5. **(C)** qPCR results of *Angpt1*, *Vegfa*, *Kdr* and *Tie1* for WT and *Apela*^{Ksp} KO mice at uI/R 21D. Scale bar = 100 μm; WT uI/R 21D, n = 9; *Apela*^{Ksp} KO uI/R 21D, n = 6; *$P < 0.05$; **$P < 0.01$; ***$P < 0.001$; ns, not significant.

Table S1. antibodies used in this study

**Antibody**	**Catalog number**	**Company**
α-Tubulin (WB)	AF0001	Beyotime Biotech
ARG2 (WB, IF)	14825-1-AP	Proteintech
WT1 (IF)	sc-393498	Santa Cruz
CD31 (IF)	65058-1-Ig	Proteintech
CD3 (IHC)	GA045229	Genetech Biotechnology
Ly6G (IHC)	551459	BD Biosciences
F4/80 (IHC)	CST70076	Cell Signaling Technology
3-NT (WB)	MAB5404	Millipore
CBR1 (WB)	Ab186825	Abcam
CBR3 (WB)	15619-1-AP	Proteintech
APJ (IF)	20341-1-AP	Proteintech

WB, Western blotting; IHC, Immuno-histochemistry; IF, Immunofluorescence.

Table S2. Primers used in this study

Gene	Forward	Reverse
M *Apela*	CGCTTAAGTTACTGCGTGCC	CAGTATCTGTCCGGCTCACC
M *Apln*	GCTGATCCCACACCCTTTCA	ACCACTCTCCCTTGGGGTTT
M *Aplnr*	CGTGGTGCTTGTAGTGACCT	GCAAACTGCCCAGCATGTAG
M *Il1b*	GCAACTGTTCCTGAACTCAACT	ATCTTTTGGGGTCCGTCAACT
M *Il6*	CACTTCACAAGTCGGAGGCT	CTGCAAGTGCATCATCGTTGT
M *Tnfα*	GACGTGGAACTGGCAGAAGAG	ACCGCCTGGAGTTCTGGAA
M *Tgfb1*	TGGCCAGATCCTGTCCAAAC	GTTGTACAAAGCGAGCACCG
M *Fn1*	GCCTGAACCAGCCTACAGAT	AGCTTAAAGCCAGCGTCAGA
M *Angpt1*	CCTTAGCATAGGGGCACACTC	TAAGGTTGCACATCCAAGCCA
M *Vegfa*	GGAGTCTGTGCTCTGGGATT	AACCAACCTCCTCAAACCGT
M *Kdr*	TTCACAGTCGGGTTACAGGC	CAGGCCAGGAGACACGTAAG
M *Tie1*	CTCTTGGGGACAGTGGGTTC	AGTCGAGGTGCAGTCAAAGG
M *Arg2*	ATTCCTTGCGTCCTGACGAG	AGCAAGCCAGCTTCTCGAAT
M *Cbr1*	CCGGTCTTGAACCACTCTCC	AAGACATGGCTGCGTGAGAA
M *Cbr3*	GGGACTTAACGTGCTGGTCA	AATGTCGAAGGGTGTTGGGT
M *Rn18s*	CTCAACACGGGAAACCTCAC	CGCTCCACCAACTAAGAACG
**Primers used for genotyping**		
Apela-flox	CTCTGAGTTCTGGCCATAGGATGTG	GGCAAACAAACAAGTTACCCTAGCC
Ksp-Cre	GCAGATCTGGCTCTCCAAAG	AGGCAAATTTTGGTGTACGG

M, *Mus musculus*

Table S3. Differential expressed genes of WT_IR1D *vs* WT ($p < 0.05$ and $|\log_2 FoldChange| \geq 1$)

Gene names	*Apela*^{ksp} read count	WT read count	$\log_2$FoldChange
Ndnf	10.90	0.00	5.90
Crtac1	8.91	0.00	5.61
Disp3	7.92	0.00	5.44
Arr3	7.46	0.00	5.35
Ccdc153	5.98	0.00	5.03
Crabp2	9.49	0.51	4.25
Adgrf3	25.76	1.54	4.09
Zfp969	57.18	4.59	3.66
Plin1	17.89	2.53	2.83
Cidec	164.05	25.37	2.70
Cbr3	1594.74	256.44	2.64
Slc6a19os	55.63	11.03	2.34
Apoa1	36.09	7.63	2.25
Crct1	68.75	16.44	2.06
Snrnp40	47.67	11.47	2.06
Cst6	20.54	4.97	2.05
Nqo1	3775.67	1076.30	1.81
Myadml2os	26.10	7.53	1.80
Tesmin	43.25	13.15	1.72
Tcf23	49.93	15.31	1.70
Cyp2a5	4624.27	1476.67	1.65
Vgf	151.56	48.54	1.64
Klk1b9	43.61	14.55	1.59
Armc12	114.46	38.25	1.59
Notumos	76.28	28.01	1.45
Plin2	11378.92	4198.11	1.44
Cbr1	7572.63	2834.48	1.42
Gnat1	134.19	50.50	1.41
Cd163	90.31	34.35	1.40
Crlf1	691.83	264.79	1.38
Aldh1a1	2222.51	853.53	1.38
Arg2	1350.64	530.55	1.35
Aldh1a7	1816.01	713.87	1.35
Hhipl1	82.89	32.71	1.34
Kcnab3	49.86	19.86	1.33
Coro1b	208.29	83.49	1.32

Slpi	113.58	46.23	1.30
Blvrb	910.22	377.70	1.27
Abhd15	104.20	43.39	1.27
Dnajb13	70.14	30.38	1.21
Cyp24a1	4393.27	1910.07	1.20
Lcn2	11300.45	4930.60	1.20
Ctla2a	157.30	68.83	1.19
Hpdl	77.39	33.97	1.19
Arhgef37	155.69	68.32	1.19
Hnf4aos	190.82	84.06	1.19
Amy1	86.66	38.21	1.19
Hilpda	80.29	35.59	1.17
Mt2	297.93	132.43	1.17
Scn1b	1033.95	460.49	1.17
Hrct1	99.92	44.69	1.16
Tshr	112.10	50.61	1.15
Fa2h	230.05	104.06	1.15
Crybg2	283.51	128.18	1.15
Mt1	8316.01	3791.49	1.13
Cd1d1	148.27	67.88	1.13
Mgst1	4403.34	2020.96	1.12
Cyp26b1	154.50	72.23	1.10
Ugt1a10	113.76	54.24	1.07
Hcn3	153.68	73.39	1.07
Tbc1d8	417.80	199.55	1.07
Egln3	497.13	238.28	1.06
Cdkn1a	1920.95	930.62	1.05
Slc25a22	1795.54	877.80	1.03
Acox1	44598.57	21916.81	1.02
Kif5a	245.16	121.67	1.01
Ndufa12	828.07	414.24	1.00
Rac2	115.39	230.03	-1.00
Slc4a11	209.06	417.69	-1.00
Igf1	79.87	159.92	-1.00
Nlrc5	135.68	271.99	-1.00
Ptprc	196.31	393.66	-1.00
Ldlr	328.09	658.15	-1.00
Traf1	45.13	90.53	-1.01
Psmb8	199.13	401.57	-1.01
Ifit3	398.63	805.99	-1.02
Tap1	331.51	673.88	-1.02

Stat1	212.69	432.70	-1.02
Brd3os	61.59	125.52	-1.03
Cd44	351.84	720.20	-1.03
Epb42	37.93	77.85	-1.04
Proz	36.74	75.59	-1.04
Dock10	228.90	472.73	-1.05
Dpp7	452.79	947.65	-1.07
Zfp239	147.29	308.68	-1.07
Pik3ap1	54.40	114.04	-1.07
Cx3cr1	181.59	380.94	-1.07
Tlr1	31.59	66.31	-1.07
Lrmp	36.97	77.86	-1.08
Themis2	59.54	126.61	-1.09
Myo1f	105.18	224.03	-1.09
Xpnpep2	58.43	124.55	-1.09
Irgm2	429.66	919.06	-1.10
F8	80.05	171.32	-1.10
Oasl2	219.00	469.73	-1.10
Psmb9	100.07	216.12	-1.11
Fcgr1	62.25	134.94	-1.12
Ltbp2	35.14	76.21	-1.12
Slc22a26	39.06	84.84	-1.12
Clstn2	29.13	63.26	-1.12
Il10ra	67.06	146.21	-1.13
Oas1a	68.83	150.23	-1.13
Ptn	39.21	85.53	-1.13
Rtp4	85.21	186.56	-1.13
Parp14	481.07	1055.76	-1.13
Pcsk9	177.35	389.81	-1.14
Bst2	326.04	717.15	-1.14
Fgd2	80.32	177.57	-1.15
Tmem150a	50.00	111.01	-1.15
H3f3c	39.47	87.99	-1.16
Stab2	28.72	64.04	-1.16
Hpgds	42.07	94.26	-1.17
Cd72	68.03	152.75	-1.17
Pou2f2	27.48	61.69	-1.17
Nfkbie	121.77	273.35	-1.17
Pvt1	23.48	53.33	-1.18
Cpxm1	100.83	229.24	-1.19
Epsti1	40.53	92.65	-1.19

Spn	40.95	94.47	-1.21
Slitrk6	28.64	66.64	-1.22
Slc34a3	168.27	391.70	-1.22
Ccr5	78.65	184.26	-1.23
Hsd3b4	93.02	219.82	-1.24
Gys2	58.36	137.94	-1.24
Trim30d	34.44	81.83	-1.25
Clec4a1	31.15	74.03	-1.25
Aspdh	195.53	466.28	-1.25
Ankrd6	106.59	255.68	-1.26
Hk3	47.07	112.77	-1.26
Gbp3	264.45	643.09	-1.28
Cxcl2	47.67	116.81	-1.29
Gatm	135.99	333.77	-1.30
Cybb	173.75	437.75	-1.33
Il1rn	55.88	142.43	-1.35
Steap4	30.39	78.30	-1.36
Cd180	27.48	70.98	-1.37
Mb21d1	25.01	64.77	-1.37
Mmp13	16.53	42.81	-1.37
Isg15	62.99	164.62	-1.39
Oas1g	21.56	56.42	-1.39
Kif11	21.39	56.65	-1.40
Sox11	18.93	50.29	-1.41
Iigp1	146.84	401.80	-1.45
Tgm7	17.48	48.56	-1.47
Lilra5	14.99	41.62	-1.47
Oas2	77.85	216.55	-1.48
Serpina3g	78.66	219.30	-1.48
Tlr2	172.57	482.08	-1.48
Clec7a	24.56	70.02	-1.51
Ifi27l2a	122.72	352.66	-1.52
Cd83	20.00	57.79	-1.53
Aif1	26.01	75.39	-1.54
Ifit1	77.81	229.28	-1.56
Fzd6	12.50	37.16	-1.57
Cd86	18.09	53.71	-1.57
Gbp5	82.13	244.78	-1.58
Gbp2	229.35	684.46	-1.58
Abcg3	15.50	46.21	-1.58
H2-DMa	130.51	390.54	-1.58

Hist1h4h	11.54	34.73	-1.59
Igtp	213.23	644.55	-1.60
Gpr65	16.44	49.74	-1.60
Ifi209	25.02	75.88	-1.60
Mfap4	22.42	68.58	-1.61
Phf11b	19.94	60.98	-1.61
Thy1	51.72	163.47	-1.66
Slc22a29	20.38	66.58	-1.71
Gvin1	34.07	113.40	-1.74
Mdk	95.46	318.44	-1.74
Gbp6	29.88	100.08	-1.74
Ifi206	10.02	33.54	-1.75
Mgl2	28.70	96.55	-1.75
Aplnr	56.30	194.85	-1.79
Clec12a	26.46	93.60	-1.82
Ifi213	16.44	58.51	-1.83
Plxnc1	19.99	71.14	-1.83
Uqcrc1	189.59	677.81	-1.84
Rpl30-ps10	58.06	209.52	-1.85
Cfi	120.58	447.22	-1.89
Aoc1	308.61	1163.38	-1.91
Ly9	12.06	45.83	-1.93
Abhd18	9.06	35.04	-1.95
Il1f6	23.90	93.37	-1.96
Cxcl5	10.40	41.11	-1.98
Serpina6	5.95	24.01	-2.01
Nat8f7	9.58	39.04	-2.03
Ppp2r3d	7.01	28.83	-2.04
Zbp1	29.94	124.65	-2.06
Lst1	8.46	35.59	-2.07
Ly86	37.90	160.65	-2.08
Foxj1	11.50	49.22	-2.10
Ccl12	11.90	51.25	-2.10
Il2rb	5.01	21.75	-2.12
Mx1	12.44	55.63	-2.16
Ms4a4c	8.51	37.99	-2.16
C4b	175.43	797.23	-2.18
Ighm	35.50	161.44	-2.19
H2-Ab1	528.70	2463.79	-2.22
Ltb	4.51	21.48	-2.25
H2-Aa	257.45	1228.93	-2.26

Apol9b	8.00	38.33	-2.26
Ccl5	23.48	112.80	-2.26
H2-Eb1	289.14	1408.56	-2.28
Il1rl1	6.56	32.02	-2.29
Cd74	978.74	4903.64	-2.32
H2-DMb2	47.17	246.55	-2.38
Ciita	47.46	250.59	-2.40
Apol9a	4.53	24.56	-2.44
Pglyrp2	5.47	29.84	-2.45
Nlrp1b	4.54	24.66	-2.45
Nek2	8.97	49.72	-2.47
Unc13c	3.48	19.49	-2.48
Cxcl10	94.26	542.34	-2.52
Slamf8	3.03	17.57	-2.54
Cd5	2.50	14.76	-2.56
Ubd	28.91	171.36	-2.57
Card11	2.50	15.55	-2.64
Haus1	5.50	36.25	-2.72
Fcrls	11.44	76.47	-2.74
Spib	2.97	21.44	-2.85
Bank1	2.00	14.56	-2.86
Ms4a4b	2.48	18.57	-2.90
Lbx2	1.49	11.37	-2.92
Amph	2.51	19.18	-2.94
Tgtp1	27.91	220.29	-2.98
Oas3	5.46	50.05	-3.19
Cd19	1.00	10.31	-3.37
Prss35	0.99	12.43	-3.64
Apela	6.47	81.54	-3.65
Podnl1	1.00	14.08	-3.82
Fam26f	0.51	9.80	-4.29
Fam19a1	0.49	10.76	-4.43
Ighd	0.51	10.86	-4.44
Tgtp2	0.51	12.91	-4.69
Cd3g	0.00	5.86	-4.99
Il17c	0.00	6.03	-5.03
Kcnh8	0.00	6.44	-5.13
Rbp7	0.00	6.85	-5.22
Iapp	0.00	7.50	-5.35
Capn11	0.00	20.28	-6.79
